# Ecologists can enable communities to implement malaria vector control in Africa

**DOI:** 10.1186/1475-2875-5-9

**Published:** 2006-02-03

**Authors:** W Richard Mukabana, Khadija Kannady, G Michael Kiama, Jasper N Ijumba, Evan M Mathenge, Ibrahim Kiche, Gamba Nkwengulila, Leonard Mboera, Deo Mtasiwa, Yoichi Yamagata, Ingeborg van Schayk, Bart GJ Knols, Steven W Lindsay, Marcia Caldas de Castro, Hassan Mshinda, Marcel Tanner, Ulrike Fillinger, Gerry F Killeen

**Affiliations:** 1 Department of Zoology, University of Nairobi, Nairobi, Kenya; 2 City Medical Office of Health, Dar es Salaam City Council, Dar es Salaam, United Republic of Tanzania; 3 Department of Zoology and Marine Biology, University of Dar es Salaam, Dar es Salaam, United Republic of Tanzania; 4 Rusinga Island Child and Family Programme/Christian Children's Fund-Kenya, Rusinga Island, Suba District, Kenya; 5 National Institute for Medical Research, Dar es Salaam, Tanzania; 6 Japan International Cooperation Agency, Tokyo, Japan; 7 National Library of Medicine, Washington, DC, USA; 8 Entomology Unit, FAO/IAEA Agriculture and Biotechnology Laboratory, Seibersdorf, Austria; 9 Laboratory of Entomology, Wageningen University & Research Centre, Wageningen, The Netherlands; 10 School of Biological and Biomedical Sciences, Durham University, Durham, UK; 11 Department of Geography, University of South Carolina, Columbia, South Carolina, USA; 12 Ifakara Health Research and Development Centre, Ifakara, United Republic of Tanzania; 13 Department of Public Health and Epidemiology, Swiss Tropical Institute, Basel, Switzerland

## Abstract

**Background:**

Integrated vector management (IVM) for malaria control requires ecological skills that are very scarce and rarely applied in Africa today. Partnerships between communities and academic ecologists can address this capacity deficit, modernize the evidence base for such approaches and enable future scale up.

**Methods:**

Community-based IVM programmes were initiated in two contrasting settings. On Rusinga Island, Western Kenya, community outreach to a marginalized rural community was achieved by University of Nairobi through a community-based organization. In Dar es Salaam, Tanzania, Ilala Municipality established an IVM programme at grassroots level, which was subsequently upgraded and expanded into a pilot scale Urban Malaria Control Programme with support from national academic institutes.

**Results:**

Both programmes now access relevant expertise, funding and policy makers while the academic partners benefit from direct experience of community-based implementation and operational research opportunities. The communities now access up-to-date malaria-related knowledge and skills for translation into local action. Similarly, the academic partners have acquired better understanding of community needs and how to address them.

**Conclusion:**

Until sufficient evidence is provided, community-based IVM remains an operational research activity. Researchers can never directly support every community in Africa so community-based IVM strategies and tactics will need to be incorporated into undergraduate teaching programmes to generate sufficient numbers of practitioners for national scale programmes. Academic ecologists at African institutions are uniquely positioned to enable the application of practical environmental and entomological skills for malaria control by communities at grassroots level and should be supported to fulfil this neglected role.

## Background

Better intervention technologies for malaria control are all clearly desirable but, taking insecticide-treated nets (ITNs) as an example, it is also clear that their cost-effective delivery remains the dominant obstacle to effective application in Africa [1]. Recent experiences with social marketing [2-5], public-private partnerships[6], decentralization [7-9] and community participation [10,11] have all provided renewed cause for optimism and shown that even the most isolated African communities can be protected from malaria through sustainable delivery mechanisms. Larval control has the potential to radically reduce malaria transmission in even the most challenging African settings [12-16] and is now being reconsidered as a complementary intervention to current priorities such as bednets and access to early diagnosis and prompt treatment [12,17,18]. Control of immature aquatic stages of *Anopheles *mosquitoes may have particular promise in urban settings where large numbers of people can be protected in a relatively small area and rural settings with focal, seasonal breeding sites [19,20]. Apart from budgetary constraints, the major obstacles to delivering these interventions to African communities appear to be a major shortfall of capacity in terms of trained personnel at all levels and a paucity of appropriate implementation structures [12,15,21].

Larval control is a labour intensive undertaking. This particularly applies to the major African malaria vectors, such as *Anopheles gambiae *sibling species, that are best tackled with rigorous searches on foot [14-16,22], because they colonize a large variety of habitats distributed widely over space and time [23-31]. In contrast, *Anopheles funestus *often requires substantial environmental manipulation or modification because this species prefers large water bodies partly shaded by vegetation that are often inaccessible by foot [13,14,31]. Larval control also requires unusual specialist skills, at all levels from community volunteer up to Ph.D. scientist [14,16,22,32-36]. As compared with delivery of bed nets or clinical services, traditional mosquito abatement methods are a predominantly outdoors activity best implemented by personnel more concerned with mosquitoes than their endophilic human victims:

*The supreme importance of getting into the field and overseeing the work must be impressed on all people in charge of malaria control. Good mosquito control means killing larvae in the field, not proud display of specimen in the office *[37]

Indeed, Fred Soper, perhaps the greatest mosquito killer in history, recognized that mosquito abatement requires a basic minimum of ecological understanding and an appetite for hard work in challenging climatic conditions [22,35]. Members of the most marginalized communities are adept amateur ecologists and enthusiastic advocates of mosquito abatement once provided with access to relevant knowledge, skills and resources. Communities represent the greatest and least exploited resource available for malaria control in Africa today. Similarly to the large-scale environmental management endeavours that America used to simultaneously rebuild its economy and roll back malaria [38], mosquito abatement may represent an equitable and productive way to utilize donor support and locally-generated funds in Africa. To mobilize this under-utilized resource requires the input of skilled personnel at all levels from the grassroots of the community up through districts, regions and national programmes.

Partnerships between academic institutions and communities have proven successful for managing not only mosquitoes and malaria [10,11,32-34,39,40], but also a plethora of different vector-borne diseases [41-46] and environmental hazards [47-50]. Students and scientists from academic departments specializing in ecological, environmental health and engineering disciplines are ideally suited to field-based research in mosquito control and possess many of the skills essential for community-based integrated vector management (IVM). IVM goes beyond simply delivering proven interventions and considers malaria transmission as a property of the local ecosystem which can be managed with judiciously chosen tools [18]. While these interventions are often technologies such as bednets or insecticides, they also include systems interventions that improve the delivery of these technologies by public health programmes or that minimize malaria transmission hazard by improving intersectoral management of the ecosystem. In the increasingly democratic context of modern Africa, systems interventions for public health or ecosystems management cannot be accomplished without the consensus and involvement of the community. The authoritarian approach applied during the colonial era, when IVM was most prominent, is no longer desirable or acceptable. Great progress has been made with horizontal community-based health programmes for delivering technologies such as drugs, diagnostics and bednets. However, with the exception of indoor residual spraying programmes in southern Africa [51,52], vertical programmes to deliver community-level mosquito control interventions have largely failed to adopt community-based approaches. By comparison, wildlife conservation [53], pollution control [50] and veterinary vector control [46] programmes have decades of experience with community-based ecosystems management and have achieved notable successes in recent years. Integrating ecologists into malaria control should greatly enhance the capacity of national malaria control programmes to deliver community-based IVM programmes. Africa needs large numbers of applied ecologists at all levels if IVM for malaria control is to be effectively and sustainably implemented on national scales. A massive and rapid expansion of this skills base can be achieved by active collaboration of academic ecologists with community-based organizations and local government.

## Methods

Community-based integrated malaria control programmes were initiated in two contrasting settings. First, a sensitization, mobilization and training collaboration was initiated between the Department of Zoology, University of Nairobi, the Rusinga Island Child and Family Programme of the Christian Children's Fund, and a marginalized rural community on Rusinga Island, western Kenya. Collaboration was established at a regular meeting of a local health coalition, intended to maximize coordination between local stakeholders. Second, the Ilala Municipal Council in Dar es Salaam, Tanzania established community-based monitoring systems for implementing mosquito control at grassroots level through street health committees. The municipal programme was then upgraded and expanded to form the spearhead of a community-based pilot-scale Urban Malaria Control Programme (UMCP) in the three municipalities of Dar es Salaam: namely Ilala, Kinondoni and Temeke. This adaptation and expansion to programmatic level [54] was assisted through academic support of the City Medical Office of Health by the Ifakara Health Research and Development Centre, the Department of Zoology and Marine Biology of the University of Dar es Salaam, and a number of international collaborators. This collaborative endeavour primarily facilitated much greater access to up-to-date knowledge and skills, notably from colleagues facing similar challenges in Kenya.

### Kenya: The rusinga island child and family programme malaria surveillance team

Rusinga Island in Suba District is an excellent example of an isolated, underdeveloped and highly disadvantaged community in western Kenya where malaria poses a major health burden in close association with increasing poverty and environmental degradation. Rusinga is home to a growing population of approximately 14,000 people with rapidly increasing levels of irrigation, urbanization of market areas, modernization in house construction, deforestation, vegetation clearance and poorly planned infrastructure development [55], leading to abundant *Anopheles *larval habitats near human settlements and endemic malaria transmission [29,56-62].

**Figure 1:**
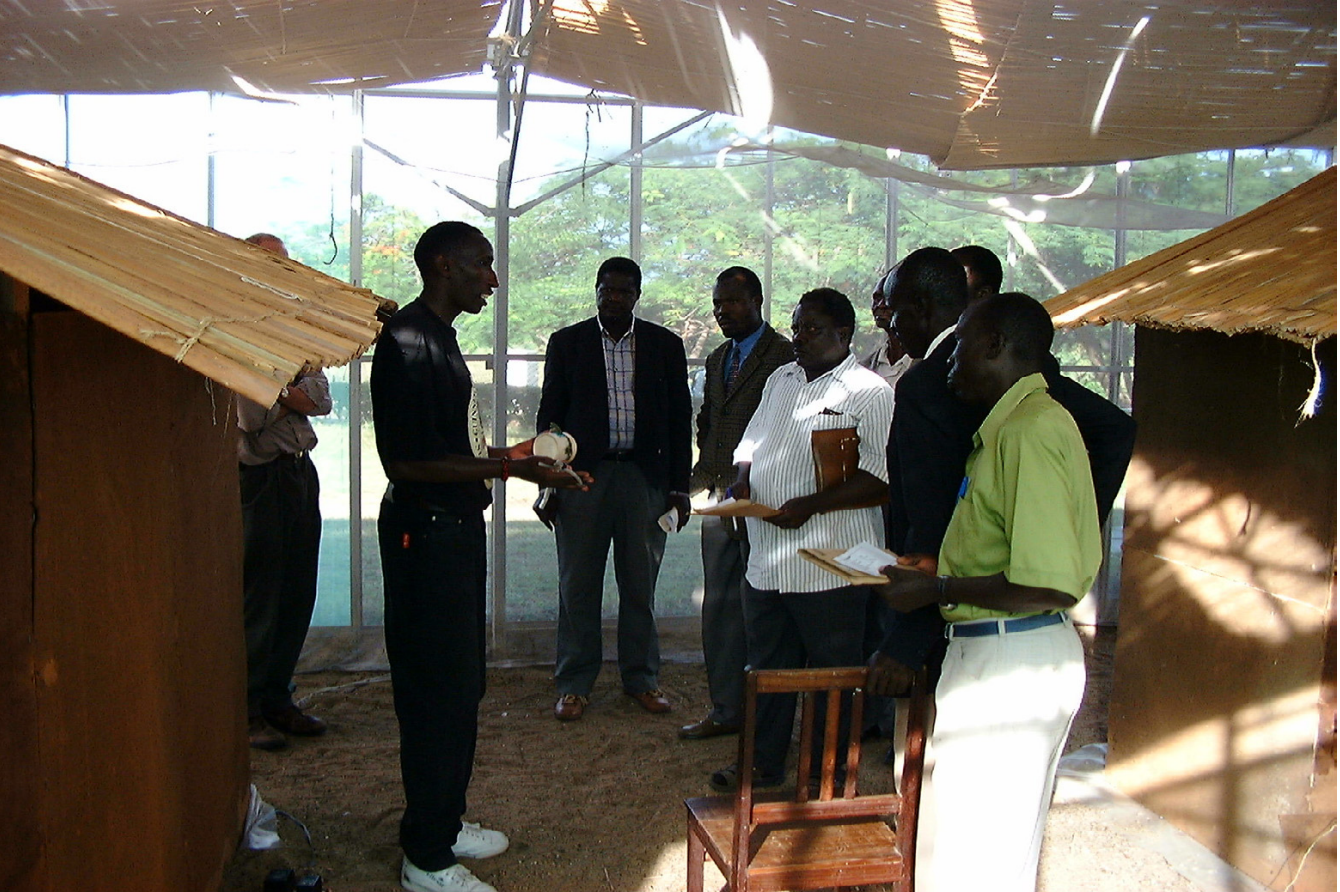
One of the authors from the University of Nairobi explains his research to community members and opinion leaders as well as government officials during an open day at the Mbita Point Research and Training Centre of the International Centre of Insect Physiology and Ecology, near Rusinga Island, western Kenya.

Mobilization of the Rusinga community in the development of their own healthcare and public health services was initially facilitated by the Rusinga Island Child and Family Programme (RICFP) of the Christian Children's Fund (CCF)-Kenya, an international non-governmental organization. The collaboration began at a health stakeholders meeting in Suba District, Western Kenya. RICFP engaged the academic partners directly and were provided, upon request, with access to the local research facilities, personnel and training through a series of open days (Figure 1). After a presentation by the academic partners on old-fashioned larval control techniques [13,15,21,38,63], RICFP/CCF invited them to train volunteers on the island. Continuous and adaptive training efforts over two years culminated in the establishment of an active malaria surveillance and control programme on the island, directly supported by the University of Nairobi, which now acts as the community's link to the international research community.

It was clear that there was an active group on Rusinga Island trying very hard to combat malaria but that their access to information and training in relation to environmental management or larviciding of malaria vectors was negligible. A good example is the commonly quoted national policy which advocates clearing of bushes for a malaria control, despite evidence that it confers no lasting protection against African anophelines [64]. Although forest clearance to expose breeding sites to sunlight can be highly effective for controlling shade-loving mosquitoes, this is very species-specific and can have the opposite effect if haphazardly applied in areas with vectors that prefer sunlit water [65,66]. Even the greatest advocates of environmental management at the height of its popularity concluded the following [65]:

we know of no instance where a small radius of clearing about houses or inhabited centres has done any good but many instances where it has done great harm

Given that the most important malaria vectors in Kenya, *An. gambiae *and *Anopheles arabiensis*, proliferate avidly in small sunlit water bodies [30,31], such clearing of vegetation typically *increases *malaria risk [25,26,67,68]. Indeed CCF has recently found that vegetation clearance is associated with increased malaria risk on Rusinga Island [56] so this is a classic example of a policy that needs to be actively reformed [47]. Broad issues of land ownership and governance in Kenya are clearly identified as targets for change in the national Poverty Reduction Strategy Paper (PRSP) [69] and Suba District's corresponding Consultation Report [70] but, like PRSPs of most tropical nations, environmental resource development strategies are conspicuously absent [47,71].

**Figure 2:**
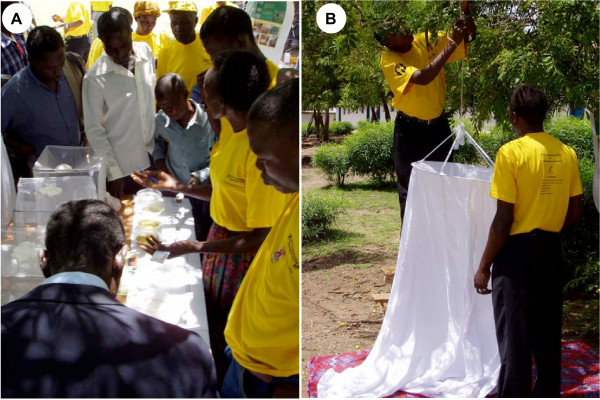
Community members demonstrate the life cycle of mosquitoes (A) and the use of practicable adult mosquito sampling tools (B) at Rusinga Island Child and Family Programme/Christian Children's Fund-Kenya, African Malaria Day, May 2004 on Rusinga Island, Western Kenya.

**Figure 3:**
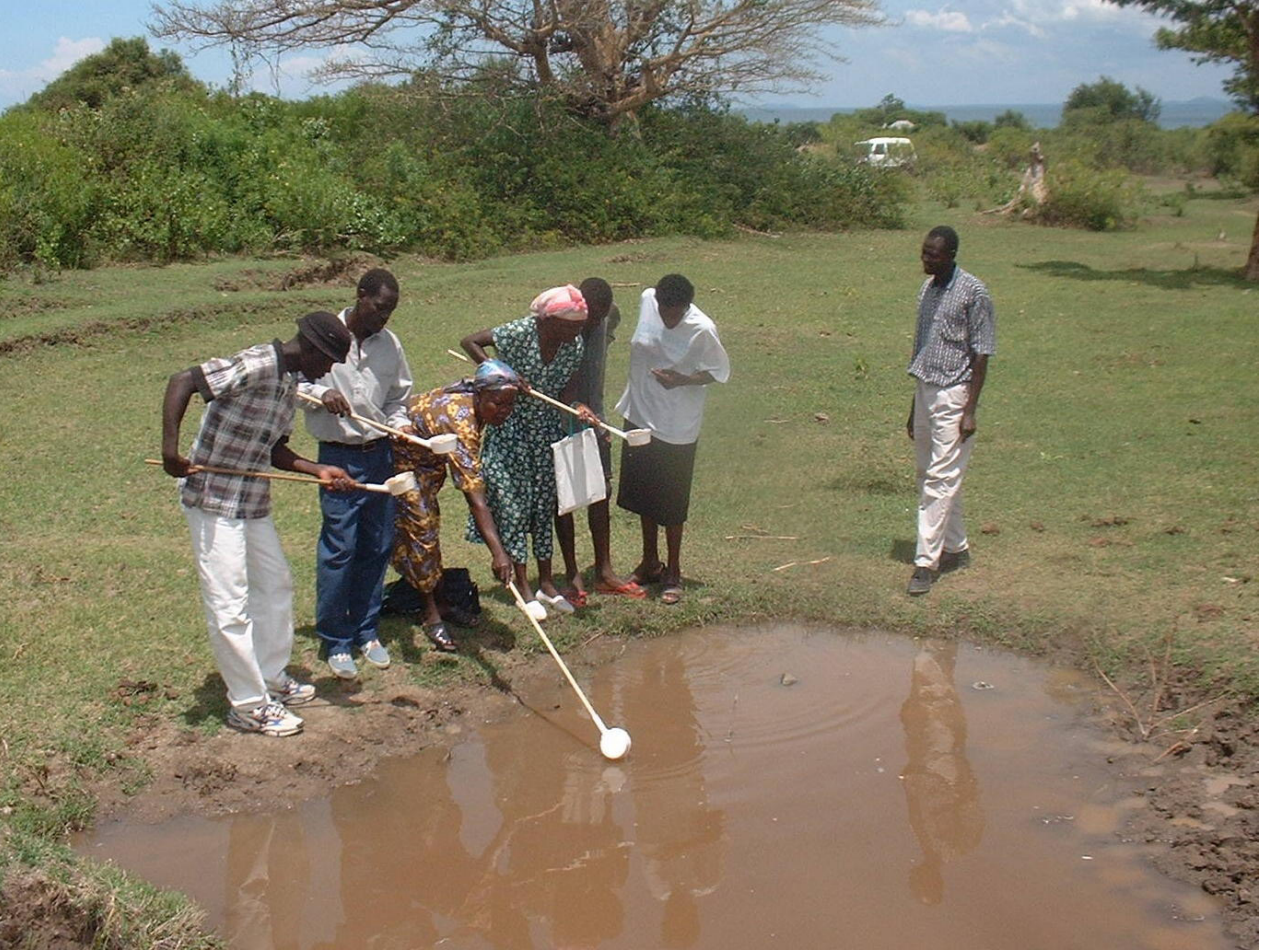
Field training of Rusinga Island Child and Family Programme community volunteers in sampling for mosquito larvae and pupae at Kaswanga, Rusinga Island, western Kenya.

At the outset of the collaboration, none of RICFP's 67 volunteers working with the community to address their malaria problems actually knew what aquatic stage mosquitoes looked like or where they could be found. Since then, RICFP/CCF has expanded it's collaborations to include local government and a number of national and international scientists through the University of Nairobi and an operational malaria surveillance based on community participation is active on Rusinga Island. Practical demonstrations using mosquito larvae and pupae taken from local habitats and allowed to emerge into cages allowed the community members to see for themselves (Figure 2). A special training course was introduced and completed which has enabled more than 70 community volunteers to distinguish *Anopheles *malaria vectors from non-vector species, to classify their larval habitats and to evaluate their productivity (Figure 3). Direct experience strongly suggests that community members find such active and tangible participation highly interesting and rewarding. It is envisaged that this will lead to rapid and sustainable changes in behaviour with knowledge transfer from children to adults and between families and friends.

The dissemination of information and adoption of appropriate malaria control activities requires all the elements of the classic theory of the Diffusion of Innovations by Everett Rogers [72]: innovation, communications channels, time and social system. The community-based larval control of malaria mosquitoes (innovation) is communicated through personal interaction during training sessions focused on learning-by-doing (communication channels). Implementation of larval control happens directly with immediate visual effects (time). Community members and health stakeholders (social system) all participate in the decision-making, planning, action and evaluation of this project that aims to enable local people to take control over their own health situation. These central elements seem to favour the process of dissemination of the innovation represented by larval surveillance and control. Additionally, this approach addresses a felt community need and is not an imposed activity but rather responds to their interest and eagerness to solve their malaria problem. The information is easy to understand and apply with low financial input. There is no obvious risk involved and results are easily experienced. It is, therefore, possible to envisage a rapid dissemination of information about community-based larval malaria control activities among members of the community.

**Figure 4:**
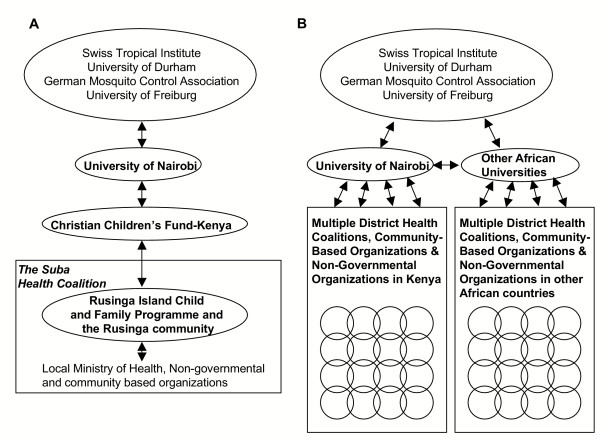
A diagrammatic of the planned institutional frameworks, as envisaged in June 2002, as a means to strengthen   malaria control capacity in Rusinga and additional malarious communities in the short (A) and long (B) term.

The experience on Rusinga bear a striking resemblance to those reported during the development of primary health care systems in nearby districts over two decades ago [73]. The Saradidi Rural Health Development Programme [74] demonstrated that parasitaemia and anaemia in pregnant mothers could be effectively tackled by community volunteers [75], who were prepared to contribute substantial time and effort with little remuneration [73]. These primary health care programmes are still operational today and it is notable that the academic support required to maintain them has been institutionalized in the form of the Teaching Institute for Community Health in Kisumu which continues to train community health workers. Although the academic side of the Rusinga Island collaboration was mainly initiated by international partners directly with the community, this is not a viable mechanism for scaling up to national levels. Therefore, responsibility for running the malaria control project has now been handed over to Kenyan scientists based at the University of Nairobi which acts as the link institute between the international partners and the community on Rusinga (Figure 4). Since then collaborative linkages have been established with a project with similar objectives but different origins in the quite different urban setting of Dar es Salaam in neighbouring Tanzania.

### Tanzania: The dar es salaam urban malaria control programme

The history of malaria control in Dar es Salaam [76] dates back more than 100 years, commencing when the area was a German possession [77-79] Larval control of mosquitoes, emphasizing environmental management has played an important role in malaria control in Dar es Salaam and other Tanzanian cities for much of the 20^th ^century [76,77]. Urban malaria control in Tanzania during the 1960s relied heavily upon larviciding and community-implemented environmental management such as drainage, filling, and other engineering works [40,77,80,81], resulting in malaria transmission that was considered to be of limited magnitude[40,77,80,81] In 1972, adverse economic conditions, combined with poor restructuring during the transition to decentralization, resulted in the deterioration of the urban health system, and chemotherapy was the only anti-malaria intervention left in place. Additionally, policy shifts at that time encouraged the citizens of Tanzania, including urban dwellers, to engage in income-generating activities, notably agriculture, which led to intensified cultivation of rice, vegetables and other crops in peri-urban areas. This increased emphasis on urban cultivation may have contributed to a ten-fold increase in the density of *Anopheles *in Dar es Salaam by the early 1980s [82].

**Figure 5:**
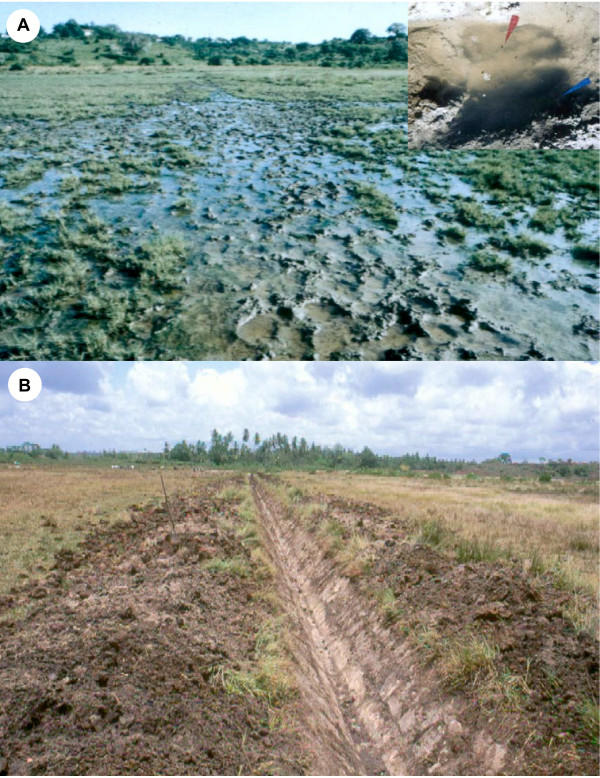
An example of successful, community implemented drain rehabilitation. A: Utofu salt marsh at the north-eastern end of Tanga, Tanzania. Insert is a hoof print harbouring a larva (blue arrow) and five pupae (red arrow) of *Anopheles merus*. Two to three boarding students were reported to die every year of high fever in Galanos High School on the hill shown on the background. 5B: One week after excavation of drains by community members assembled by the school.

In 1987, the Government of Japan, through the Japan International Cooperation Agency (JICA), provided external assistance for urban malaria control in Dar es Salaam and Tanga to reinitiate mosquito abatement in these urban centres. This programme was successful in terms of community-implemented environmental management, particularly the rehabilitation of drainage infrastructure (Figure 5) [76,83] but national ownership and capacity were not sufficiently developed by this programme to achieve sustainability and the programme ended in 1996. Although valuable lessons about working with the community were learned [84], the project retained a centralized structure as a vertical programme directly under the Ministry of Health and was poorly adapted to working in a participatory fashion, even to the extent that indoor residual spray programmes had to be abandoned in favour of bednet distribution because residents refused access to their houses [76,83].

**Figure 6:**
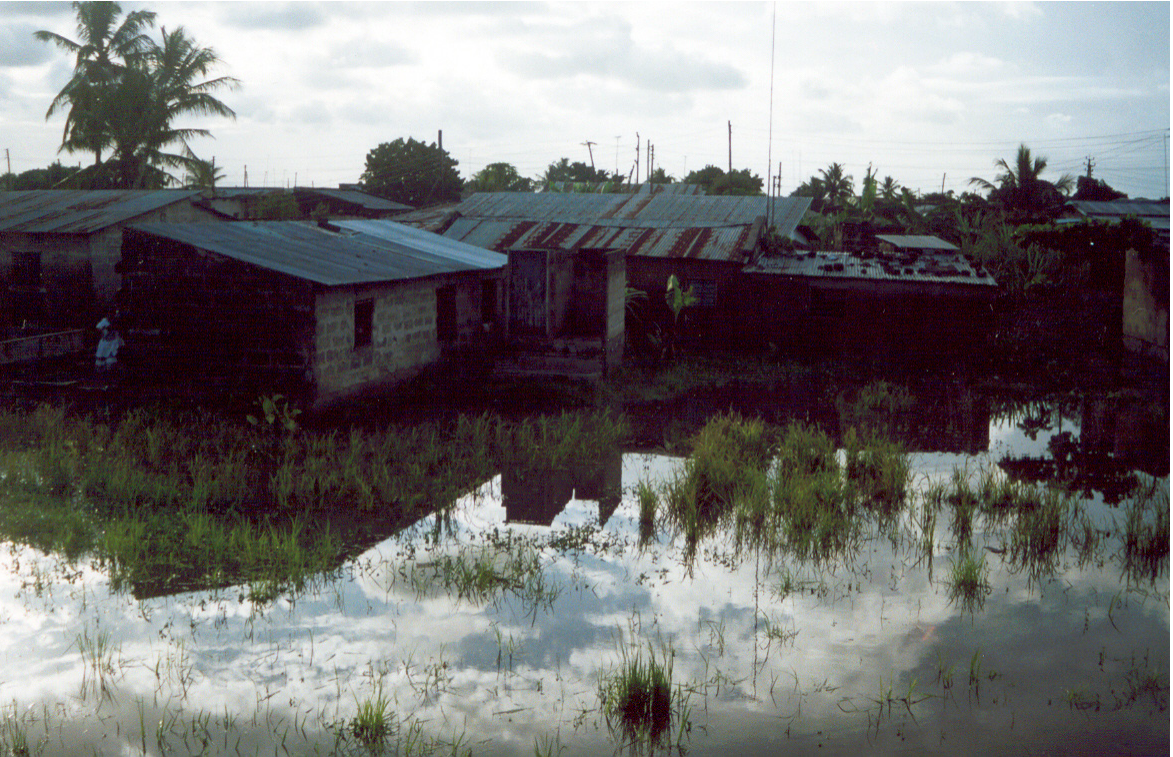
Examples of flooded areas in the poorly planned settlements of Vingunguti in Ilala municipality, Dar es Salaam, Tanzania.

**Figure 7:**
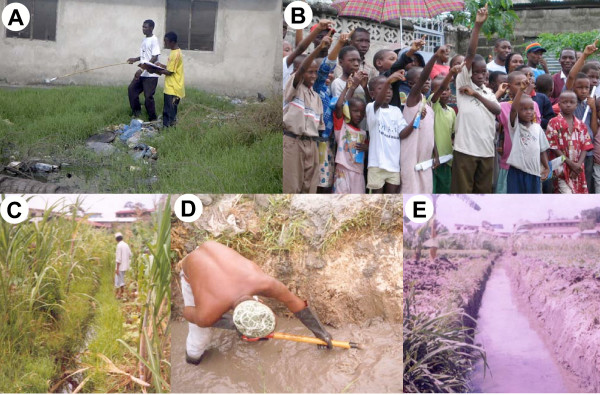
Examples of community-based malaria surveillance and control in Dar es Salaam. A. Community Own Resource Persons mapping and characterizing mosquito breeding sites in Vingunguti ward, Ilala Municipality in May 2004. B. Children participate in a quiz about malaria and mosquitoes in Mchikichini ward, Ilala Municipality, in April 2004. C. Extensive agricultural breeding sites associated with neglected drains adjacent to Ruihinda Primary School (background), Kigogo Ward, Kinondoni Municipality, in June 2001. D. Kinondoni Municipal Council workers rehabilitate the drain to lower the water table in August 2001. E. The same plots in September 2001.

In recent years, the ten-year Dar es Salaam Urban Health Project [8,85,86], has considerably strengthened healthcare and public health infrastructure within the city through a decentralized health system [7], which is also ideal for delivering community-based vector control interventions [15,34,39,87]. This degree of autonomy enabled Ilala Municipal Council to independently conceive, fund and implement a community-based mosquito surveillance programme as an entirely local initiative in early 2002. Supplementing ongoing programmes for social marketing of ITNs and improved access to effective diagnosis and treatment [88], teams of community members were recruited to map and characterize the extensive breeding sites provided by intensive urban agriculture and poorly planned settlement (Figure 6) which abound in Dar es Salaam [76,89] and many other African cities [19,20,90-93]. Weekly surveys for larval and adult mosquitoes were piloted in 7 of the 22 wards of Ilala Municipality as a preliminary step towards IVM by larviciding and environmental management. Furthermore, all three municipal councils had actively promoted a variety of locality-specific community-based environmental management schemes such as trench digging and collection of solid waste refuse to prevent obstruction of such drains (Figure 7). In June 2003 the City Medical Office of Health (CMOH) convened a stakeholders meeting for all concerned with malaria in Dar es Salaam, and invited a consortium of national and international scientific partners, including those involved with the Rusinga Island project in Kenya. Consensus was reached by all stakeholders that control of aquatic-stage mosquitoes, particularly source reduction through environmental management, was highly desirable and should be added to the national priority interventions to complete an integrated suite of interventions for the city. With support from the national and international academic partners, the City Medical Office then formulated a detailed implementation plan for evaluating the incremental effectiveness of larval control delivered by community members in 15 of the 73 wards of Dar es Salaam, in addition to the existing national programme interventions. Importantly, this demand-driven implementation plan for the UMCP built upon the community-based systems developed in Ilala, in which responsibility for routine mosquito surveillance is delegated to individual community members, known as Community-Owned Resource Persons (CORPs), appointed and managed through Street Health Committees.

The consensus view of the stakeholders was that it was essential to strengthen the capacity of research and training institutions in Tanzania, within the initial pilot period of the programme, to build the necessary academic support base required to maintain the essential skills base on an indefinite basis. The primary national institutes involved included the Ifakara Health Research and Development Centre and the Department of Zoology and Marine Biology at the University of Dar es Salaam. These institutes were identified as not only supporting institutes but also as targets for strengthening training capacity so that the quantity and quality of applied ecologists available at undergraduate and graduate levels for recruitment to mosquito control programmes could be improved. Although this community-academic collaboration differs considerably from Rusinga in its setting and origins, the partnership has already begun to address capacity deficits by bringing expertise in operational mosquito control to the Municipal and City teams, including over 100 CORPs (Figure 7). As on Rusinga, the academic partners observed that standards of training and practice were initially quite poor within the existing municipal health teams who had had no expert training or access to current training material relating to mosquito abatement.

In November 2003, the Centre for Enhancement of Effective Malaria Interventions (CEEMI [94]) of the National Institute for Medical Research (NIMR) and the National Malaria Control Programme (NMCP) of Tanzania conducted a sensitization workshop with the country's Members of Parliament (MPs) aimed at soliciting for their support in promoting the use of ITNs in line with the National Medium-Term Strategic Plan [88]. While the MPs generally endorsed the interventions prioritized by the national plan, particularly the use of ITNs, many requested reconsideration of mosquito abatement, which many of them remembered from previous programmes, and greater support for such activities. Immediately afterward, at a meeting held in Dar es Salaam and sponsored by Tanzania NGOs' Alliance Against Malaria (TaNAAM), it became apparent that, NGOs and CBOs, including scouts and girl guides, were willing and ready to undertake many tasks pertaining to malaria control at community level but lacked the necessary skills and coordination mechanism. Thus, although the local circumstances and mode of interaction proved quite different to the Rusinga scenario, the message from the community to ecologists, health scientists and policy makers was clear: the people of Tanzania requested help from their national institutes to develop mosquito abatement programmes similar to those they have experienced in the past (See references [76,80,95] for reviews).

## Discussion

The Rusinga Island project has been a rewarding and enjoyable experience for all partners because the actual nature of the collaboration was determined by the community and the academic scientists were integrated into their activities rather than *vice versa*. Similarly, the initial reimplementation of mosquito abatement activities in urban Dar es Salaam was an entirely local initiative that was conceived, planned, implemented and funded by the Ilala Municipal Council in collaboration with community representatives right down to the level of street chairpersons and ten-cell unit leaders. As the community of Tanzania make their voices heard through grassroots community-based field workers, CBOs, NGOs and elected parliamentary representatives, academia has been challenged to increase the level of research and training support it provides to district-level vector control initiatives.

Many communities across Africa are just as active and determined as the residents of Dar es Salaam and Rusinga to implement malaria control through their own CBOs and NGOs but have had little access to the basic information and training which would enable them to do so. Unlike the parasites which actually cause malaria illness and death, the mosquitoes that carry them are readily visible, distinguishable and vulnerable to the community members upon whom they feed. Malaria-endemic Africa is home to 521 million community members [96], many of whom could be engaged to apply low technology interventions at minimum cost given the basic skills to dip for larvae, trap adult mosquitoes, distinguish *Anopheles *and spray non-toxic bacterial insecticides. For now, the evidence base for integrated malaria vector management is limited to a few dusty books that precede the advent of modern epidemiological tools. Direct experience with mosquito abatement in Africa is rapidly fading from living memory. Also, the implementation of larval control through community-based systems remains an unproven approach for malaria prevention in Africa. While a number of efficacy and effectiveness trials of IVM are underway across Africa, no clear consensus about how best to achieve comprehensive or targeted coverage yet exists [97]. The existing expertise of national academic institutions to support IVM, as well as the evidence base to justify it, are not yet sufficient for inclusion in national programmes. Community-based IVM should therefore only be practiced as an operational research activity until sufficient evidence and capacity is present to enable implementation at national level. Because researchers can never directly support every community in Africa, community-based IVM approaches should be incorporated into undergraduate teaching programmes in the near future so that sufficient numbers of practitioners can implement effective programmes on national scales. Mobilization of communities across Tanzania, Kenya, or any other country in Africa to implement community-based larval control will require large numbers of competent mosquito ecologists at diploma and bachelors' level, as well as the masters and doctoral graduates who will train and direct them. In order to effectively teach community-based IVM in the future, academic ecologists need to rapidly engage in relevant operational research programmes so that knowledge of this challenging topic goes beyond reading from outdated books to include relevant, contemporary experience. The close linkage between research and control activities was a key factor in the success of the Onchocerciasis Control Programme [42], as well as malaria control programmes from the first half of the century [98]. The same integration needs to be achieved between community-based malaria control and vector ecology research in Africa. Academic ecologists engaged in entomology, engineering, agricultural, zoology or environmental sciences should be more actively supported as partners for Rolling Back Malaria.

## Conclusion

It remains to be seen whether community-based IVM can be cost-effective and sustainable so it remains an operational research activity until sufficient contemporary evidence is provided. Ecologists at African academic institutions are ideally positioned to develop the evidence base and scale up capacity for community-based implementation of practical environmental and entomological malaria control skills with technologies that already exist and are readily available. They should now be supported to fulfil this neglected role.

## Competing interests

Both the Rusinga and Dar es Salaam projects have been supported financially by Valent Biosciences Corporation, a commercial manufacturer of microbial larvicides.

## Authors' contributions

The views expressed in this opinion article reflect the outcome of extensive discussions amongst the authors, and with the communities they work with in Kenya and Tanzania. The manuscript was drafted by GFK in consultation with all the other authors who participated in the activities described and agree with the final submitted form.
